# Near-Infrared Imaging of Glymphatic Clearance in a Pre-Clinical Model of Repetitive Closed Head Traumatic Brain Injury

**DOI:** 10.1089/neur.2024.0128

**Published:** 2025-01-30

**Authors:** Eleftheria Michalaki, Alexis N. Pulliam, Pooja M. Datta Roy, J. Brandon Dixon, Michelle C. LaPlaca

**Affiliations:** ^1^ George W. Woodruff School of Mechanical Engineering, Georgia Institute of Technology, Atlanta, Georgia, USA.; ^2^ Parker H. Petit Institute for Bioengineering and Bioscience, Georgia Institute of Technology, Atlanta, Georgia, USA.; ^3^ Wallace H. Coulter Department of Biomedical Engineering, Georgia Institute of Technology and Emory University, Atlanta, Georgia, USA.

**Keywords:** brain clearance, cerebrospinal fluid, cervical lymph nodes, closed head repetitive impact, glymphatic system, lymphatic system, mild traumatic brain injury, near-infrared imaging, traumatic brain injury

## Abstract

Traumatic brain injury (TBI) is a major health disorder for which there are few treatments. The glymphatic system is the brain’s inbuilt lymphatic-like system that is thought to be responsible for clearing waste products from the brain to the lymph nodes. Although there is evidence that glymphatic drainage is crucial for brain homeostasis, its role in TBI pathogenesis remains elusive. Here, we investigated how glymphatic clearance is altered following TBI in rats using real-time non-invasive imaging. Twenty-four hours following repetitive closed-head TBI or sham conditions, we injected infrared dye intraventricularly and used near-infrared (NIR) imaging to quantify signal intensity, intensity over time, and appearance time of NIR dye in different brain regions. TBI yielded a lower NIR signal and lower rate of NIR dye change in the lateral ventricle and surrounding parietal cortex compared with sham conditions, indicating reduced cerebrospinal fluid perfusion. NIR dye appearance took significantly longer to reach the anterior regions of the brain, while perfusion to the posterior of the brain was faster in TBI compared with sham animals. Aquaporin-4 (AQP4) expression was reduced 24 h after TBI across all cortical regions examined in the posterior of the brain and in the ventral cortex at all coronal levels, suggesting a complex relationship between AQP4 and glymph function. Furthermore, NIR imaging revealed that NIR dye was detectable in the cervical lymph nodes (CLNs) of sham animals but not in TBI animals, yet there was evidence of blood accumulation in the CLNs of TBI animals, suggesting that TBI-related extravascular blood is removed through the glymph system. These data indicate that TBI disrupts normal brain efflux kinetics and reduces glymphatic drainage to the CLNs, demonstrating that restoring glymphatic function may be a promising therapeutic target.

## Introduction

Traumatic brain injury (TBI) is a leading cause of death and disability, with nearly 70 million people worldwide estimated to sustain a TBI each year.^1,2^ The paucity of treatments is, in part, due to heterogeneity and complexity of injury mechanism and secondary injury cascades.^3,4^ TBI is associated with a prolonged neuroinflammation, heightened oxidative stress, altered cell signaling, and cell energy crises, among other processes.^4–6^ Cellular damage and waste products are detected and partially cleared by resident microglia and recruitment of peripheral immune cells,^7^ encompassing a presumptive role for meningeal lymphatics in the secondary injury response.^8–10^

The connection between the lymphatics and the brain is thought to involve a fluid network of the cerebrospinal fluid (CSF), paravascular spaces, and interstitial fluid (ISF), termed the glymphatic system.^11,12^ The glymphatic system plays a role in processing CSF/ISF exchange and thought to be the dominant drainage conduit for waste products and macromolecules from the brain to the cervical lymph nodes (CLNs).^11,13,14^ The normal flow path for glymph fluid involves circulating CSF that enters the brain from the subarachnoid space to the periarterial spaces, mixing with ISF and traveling along the perivenous space (collectively referred to as paravascular here). Glymphatic efflux from the brain is through the cribriform plate,^15,16^ arachnoid granulations, nasal and meningeal lymphatics, and cranial nerves into the deep CLN (dCLN) and superficial CLN (sCLN).^14,17–20^

The glymphatic system has been shown to play a role in clearance of proteins following TBI, such as tau,^21^ glial fibrillary acidic protein, and S100β.^22^ An increase in glymphatic influx and decrease in glymphatic efflux in deep brain regions, but not in the cortex, was seen 1 day after repetitive TBI.^23^ TBI has also been shown to cause a reduction in overall glymphatic flow (i.e., influx and efflux) across different brain regions 10 months following TBI.^24^ Although there is evidence that glymphatic clearance is involved in maintaining brain homeostasis, deficiencies in TBI and other neurological diseases remain only partially understood.^25–28^

There is an ongoing debate about dominant contributing factors to the driving force for glymphatic flow.^29^ Therefore, a better understanding of glymphatic kinetics may provide new therapeutic targets for restoring glymphatic function after TBI and other neuropathologies.^30^ Here, we investigate how glymphatic/lymphatic function is altered following experimental TBI. We used repetitive closed-head impact and near-infrared (NIR) imaging to quantify post-TBI intracerebral CSF movement and efflux from the brain to the cervical lymphatics. We postulate that TBI affects brain fluid flux kinetics and slows glymphatic efflux to the CLNs, which may, in turn, limit effective clearance of cellular waste products, exacerbating TBI-induced damage.

## Materials and Methods

### TBI model

Male Sprague Dawley rats 250–400 g (Charles River Laboratories, Wilmington, MA) were used in accordance with the guidelines set forth in the Guide for the Care and Use of Laboratory Animals (U.S. Department of Health and Human Services, Washington, DC, USA, Pub no. 85-23, 1985) and were approved by the Georgia Institute of Technology Institutional Animal Care and Use Committee (protocol #A100188). A modified controlled cortical impact (CCI) device (Pittsburgh Precision Instruments, Pittsburgh, PA) was utilized to induce repetitive closed-head injuries to the dorsal surface of the head (*n_TBI_* = 12; *n_Sham_* = 14). Rats were anesthetized with isoflurane (4% induction, 1–2% maintenance) and positioned under the pneumatic piston (fitted with a 1-cm diameter silicone tip; Renovators Supply Manufacturing, Erving, MA) and atop a 1-inch thick foam support (ethylene-vinyl acetate; McMaster-Carr, Douglasville, GA, 86095K46). Thirty seconds after removal of isoflurane the piston impacted the head (velocity 5 m/s, dwell time 50 msec) with 3 impacts at 2-min intervals (head displacements = 5, 2, 2 mm). The repeat closed-head injury model^31–34^ using the CCI device was developed to model sports-related injuries—boxing, soccer, football, and other contact sports—where an athlete may receive multiple concussive and/or subconcussive impacts during training or match play.^35–39^ Rats were placed on maintenance isoflurane between impacts for 1.5 min. Following the final impact, righting time was recorded and animals were monitored until fully mobile. Impacted animals with righting time below 4 min were excluded from the study as they were not qualified as injured. Sham animals in our model have a righting time under 3 min to qualify as sham controls.^31,32^ The average righting time of the TBI animals included in the study was ∼6.4 ± 2.1 min.

### Tracer infusion

Rats were anesthetized with ketamine/dexmedetomidine (40:0.13 mg/kg, i.m.) 24 h post-TBI. The skull was exposed, and a small hole (∼1 mm) was drilled 1.3 mm lateral and 1.6 mm posterior to the bregma. NIR dye (LI-COR IRDye 800 CW [929–70021; LI-COR Biosciences, Lincoln, NE], 20 kDa methoxy polyethylene glycol amine [07966-250MG; Sigma Aldrich, St. Louis, MO]) was infused (total volume = 7 µL, rate = 1 µL/min; 53313; Stoelting Co) into the right lateral ventricle (3.6 mm ventral from the skull) using a Hamilton needle (Gastight #1701; Hamilton Company, Reno, NV), which was left in place for 5 min to avoid pressure-induced backflow.

### NIR imaging procedures

**FIG. 1.**
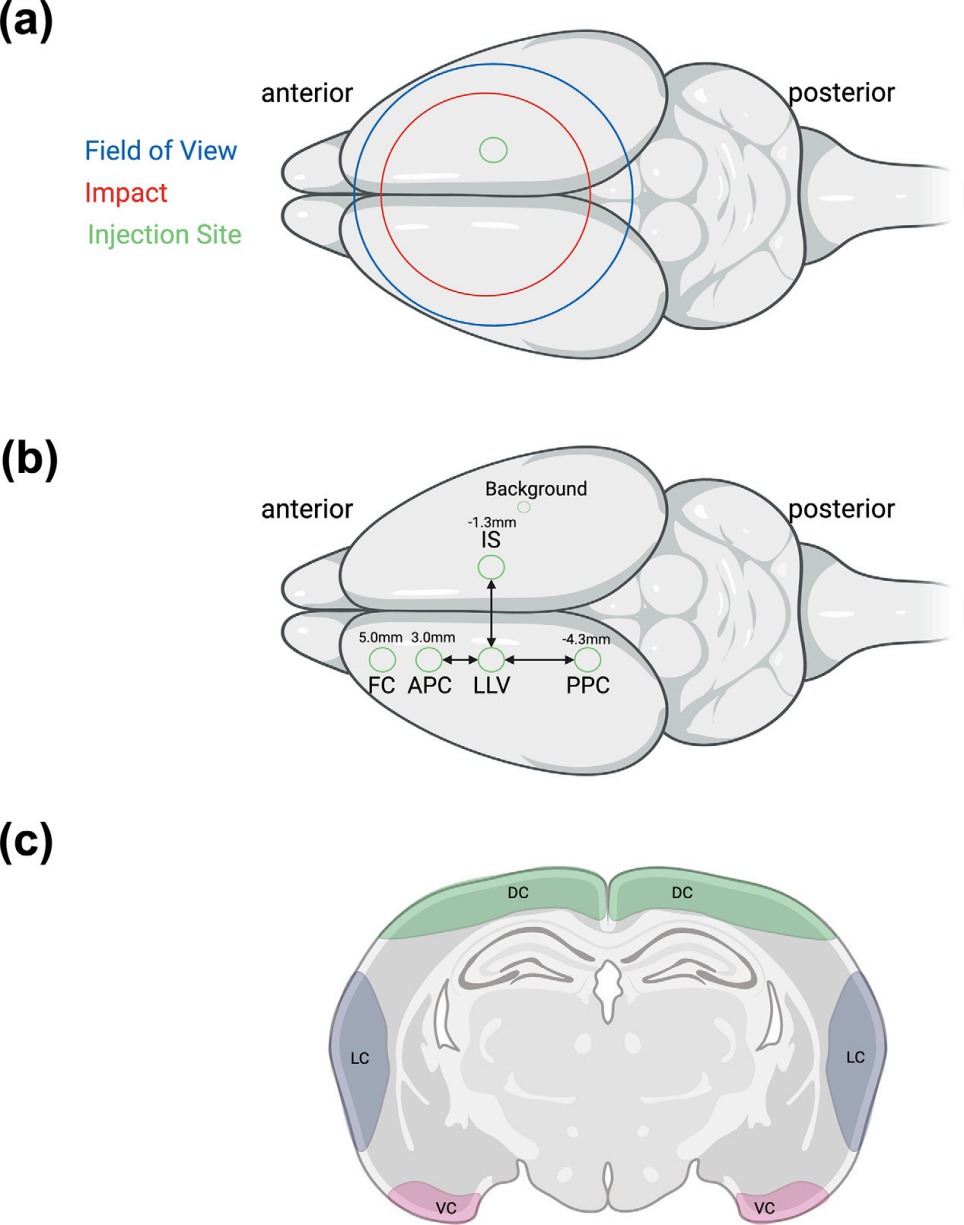
Anatomical reference regions of interest (ROIs) for injury and quantification of cerebrospinal fluid (CSF) kinetics and aquaporin-4 immunohistochemistry. Dorsal view of a rat brain to highlight **(a)** the field of view (blue circle) during near-infrared (NIR) imaging and injection site (green circle) with respect to the position of the traumatic brain injury (TBI) impact (red circle). **(b)** ROIs for NIR imaging, from anterior to posterior: frontal cortex (FC), anterior parietal cortex (APC), left lateral ventricle (LLV), injection site (IS), and posterior parietal cortex (PPC). The background region was used to calculate the noise. **(c)** ROIs for immunohistochemistry: dorsal cortex (DC), lateral cortex (LC), and ventral cortex (VC). Illustrations created using BioRender.

TBI and sham animals were imaged using NIR with identical imaging procedures beginning 15 min after tracer infusion. NIR imaging began 15 min after tracer infusion and continued for 60 min. Images were taken with a customized imaging system consisting of a Lambda LS Xenon arc lamp (LB-LS; Sutter Instrument, Novato, CA), an Olympus MVX-ZB10 microscope (Olympus Corporation, Japan), a 769 nm bandpass excitation filter (49 nm full-width half maximum; FWHM), an 832 nm bandpass emission filter (45 nm FWHM), and an 801.5 nm longpass dichroic mirror. Images were acquired with Photometrics Evolve Delta 512 EM-CCD (Teledyne Photometrics, Tucson, AZ) as previously described.^40^ The field of view was centered on the exposed skull (Fig. 1a). NIR images of dynamic tracer intensity were captured at a rate of 1 frame per min with a 250 msec exposure time for a total of 60 min. After the completion of brain NIR imaging, the neck was shaved, and sCLN were imaged.

### NIR image analysis

CSF perfusion was quantified by analyzing the spatial and temporal intensity of NIR fluorescence. Images were imported into ImageJ^41^ and scaled. Regions of interest (ROIs, 2-mm diameter) in the exposed skull images were defined (Fig. 1b). A 2.6-mm line was drawn from the injection site (IS) to the left lateral ventricle (LLV) region. From the perimeter of the LLV circle and 2.6 mm anterior of the LLV, the anterior parietal cortex (APC) area was designated, and the posterior parietal cortex (PPC) area was defined 2.6 mm posterior to the LLV. The frontal cortex (FC) was identified as the most anterior portion of the brain in the imaging field at approximately +4.5 mm rostral to bregma. Finally, the corresponding background was quantified by defining a smaller circle (diameter = 0.1 mm) away from the ROIs.

CSF kinetics in the cerebral extravascular space was described by four measurements: (1) normalized intensity, which is the average NIR fluorescence signal intensity of the specific ROI divided by the average intensity of the background ROI in the corresponding animal and point in time^40^; (2) intensity/time, which is the slope of a linear fit of the average intensity over time for the designated ROI; and (3) appearance time, defined as the time that the fluorescence intensity of the NIR signal reaches 10% above the initial background fluorescence intensity measurement at *t* = 0 for a particular ROI, thus describing the influx time from injection to each ROI.

### LN and brain harvest

Following NIR imaging, animals were transcardially perfused with phosphate buffer (0.1 M, pH 7.4), followed by 4% paraformaldehyde (P6148; Sigma Aldrich) in phosphate buffer. After perfusion, the sCLNs and dCLNs, axillary LNs (negative controls), and brains were harvested. To dissect the CLNs, a rostral-caudal incision was made in the ventral neck area and sCLNs and dCLNs were harvested bilaterally (2–6 nodes per site). Following decapitation, the brains were removed.

### Immunohistochemistry

Brains were post-fixed in 4% paraformaldehyde (Sigma Aldrich) for 24 h at 4°C and coronally sectioned with a vibratome (Leica VT1000; Wetzlar, Germany) at 100 µm. Brain sections were selected from the following anterior/rostral to posterior/caudal levels that correspond to the NIR ROIs, namely, FC, bregma +4.3 mm; left lateral paraventricular tissue, LLV, bregma +1 mm; and PPC, bregma −4.3 mm. Within each section at each level, we examined three cortical ROIs: dorsal cortex (DC), lateral cortex (LC), and ventral cortex (VC). These regions were chosen based on previous studies showing the greatest change in glymphatic flow after injury.^42^ All sections were incubated in anti-aquaporin-4 (AQP4) primary antibody (Alomone Labs, Limerick, PA 1:500, Cat. AQP4-004, RRID: AB_2039734) overnight then placed in anti-rabbit secondary antibody (ThermoFisher Scientific, Waltham, MA, 1:500, Cat. T2767, RRID: AB_221655). All brain sections were counterstained with DAPI (ThermoFisher, 1:1000, Cat. D1306, RRID: AB_2629482) and imaged using confocal microscopy (Zeiss LSM 700 Confocal Microscope System; Zeiss; Oberkochen, Germany) at 20× magnification. Image processing was done using FIJI ImageJ^43^ (https://imagej.net/Fiji) to obtain the corrected total image fluorescence by subtracting the product of the selected area and mean fluorescence background from the integrated density.

### Histology

Harvested LNs (sCLNs, dCLNs, and axillary LNs) were post-fixed in 4% paraformaldehyde (50-980-487; ThermoFisher) at 4°C for 24 h, embedded in OCT medium (23-730-571; ThermoFisher), and then frozen at −80°C. LN samples were cryosectioned (Cryostar NX-70; ThermoFisher) into 10-µm sections and mounted on poly-L-lysine-coated slides. Hematoxylin and eosin (H&E; ab245880; Abcam, Cambridge, United Kingdom) staining was performed using a Leica Autostainer XL (ST5010; Leica Biosystems). H&E slides were imaged using a Zeiss Z1AxioObserver microscope (Z1; Zeiss), and images were taken at 60×.

### Data analysis

To compare the effect of TBI on CSF/glymph perfusion, an unpaired *t*-test with Welch’s correction and Robust regression and OUTlier removal (ROUT) method to identify and remove outliers were used.^44^ Immunohistochemistry was analyzed by measuring total fluorescence intensity and compared between groups by nested one-way ANOVA with Tukey’s multiple comparisons test. Data were analyzed using GraphPad Prism 8. Reported *p* values are multiplicity adjusted to account for multiple comparisons. For all cases, significance was defined as *p* < 0.05 (*), *p* < 0.01 (**), *p* < 0.001 (***), or *p* < 0.0001 (****).

## Results

### Closed-head mild TBI reduces intracerebral CSF/glymph movement

**FIG. 2.**
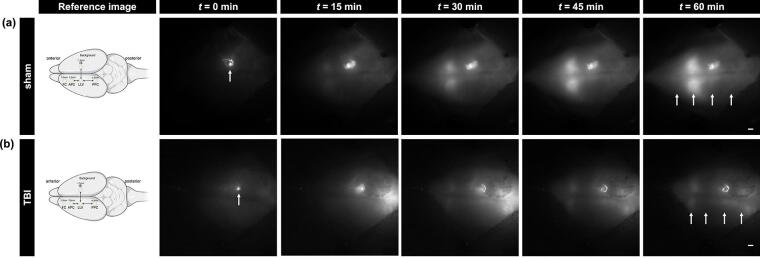
Closed-head traumatic brain injury (TBI) affects near-infrared (NIR) efflux kinetics. Representative NIR imaging through the skull allows visualization of the NIR dye. At *t* = 0 min, white arrows depict the injection site of NIR dye 15 min post-injection (*t*_0_ = 15 min). Live NIR imaging demonstrates the presence of different efflux kinetics between sham and TBI animals. At *t* = 60 min, the various regions of interest (ROIs) take up the NIR dye. White arrows point at the different ROIs as seen in the schematic; from anterior (left) to posterior: frontal cortex (FC), anterior parietal cortex (APC), left lateral ventricle (LLV), injection site (IS), and posterior parietal cortex (PPC). Scale bar = 1 mm.

We found that all intracerebral ROIs (see Fig. 1b, FC, APC, LLV, and PPC) take up NIR dye (after *t* = 0), but NIR dye moved to different ROIs at different rates for sham and injured animals during the 60 min of live imaging, as indicated by the different levels of fluorescence signal for a given time point (Fig. 2). In sham animals, dye intensity was higher in areas anterior of the IS, namely, FC and APC, compared with TBI animals relative to the PPC/caudal areas of the brain (Fig. 2a). Despite the regional differences, overall NIR signal intensity was lower in injured animals relative to sham animals (Fig. 2b).

### Closed-head TBI contributes to altered kinetics and pathways for CSF/glymph movement

**FIG. 3.**
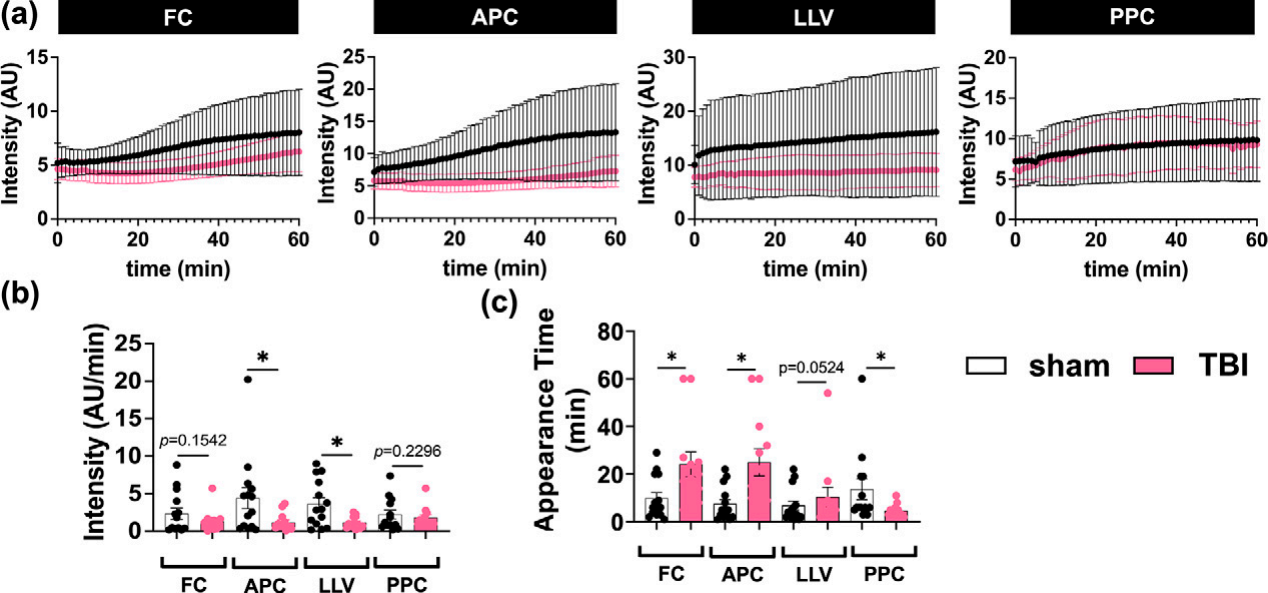
Use of near-infrared (NIR) imaging to quantify intracerebral glymph perfusion in sham and traumatic brain injury (TBI) animals. Quantification of: **(a)** NIR intensity (AU, normalized to background); note that the *y*-axis value ranges are different among the brain levels examined, in order to better visualize the patterns between TBI and sham for each level, **(b)** intensity/time (AU/min), and **(c)** appearance time. Each data point corresponds to an independent animal (*n_sham_* = 14 and *n_TBI_* = 12) and error bars indicate the corresponding standard error of the mean. Solid lines above the plots indicate a comparison for significance using an unpaired *t*-test with Welch’s correction, *p < 0.05. Regions of interest (ROIs): frontal cortex (FC), anterior parietal cortex (APC), left lateral ventricle (LLV), and posterior parietal cortex (PPC).

We found that NIR fluorescence intensity for FC, APC, and LLV was lower in TBI animals compared with sham, implying that CSF transport is impaired after TBI (Fig. 3a). Relative to the anterior and center of the brain, posterior brain areas in TBI animals exhibited less difference in tracer intensity from sham conditions in the posterior cortex, indicating that CSF perfusion is relatively faster, preferentially moving, or stagnating toward the posterior area of the brain following TBI.

Rate of NIR dye change is an indication of CSF flow rate. We showed a significantly lower rate of NIR tracer change in the APC and LLV areas of TBI brains compared with sham, implying slower CSF perfusion out of the IS and to the most proximal anterior region following TBI (Fig. 3b). The most anterior (FC) and most posterior (PPC) ROIs examined showed no differences between TBI and sham (Fig. 3b).

TBI significantly increased the appearance time in the anterior areas of the brain, namely, FC and APC (*t_FC_* = 24 min and *t_APC_* = 25 min, respectively), compared with sham (*t_FC_* = 10 min and *t_APC_* = 7 min, respectively) (Fig. 3c). In the most posterior region examined, PPC, the appearance time was significantly shorter in TBI animals (*t_PPC_* = 5 min) compared with sham (*t_PPC_* = 14 min) (Fig. 3c), suggesting relatively faster flow. Time of dye appearance in the LLV did not differ between groups (Fig. 3c), not unexpectedly given that the IS was in the right lateral ventricle. Together, these results suggest differences in glymphatic influx across the anterior to posterior axis of the brain, and potentially the presence of different glymphatic pathways, flow directions, or flow rates within the brain following TBI compared with control conditions.

### Closed-head TBI leads to loss of AQP4 expression in the posterior brain

**FIG. 4.**
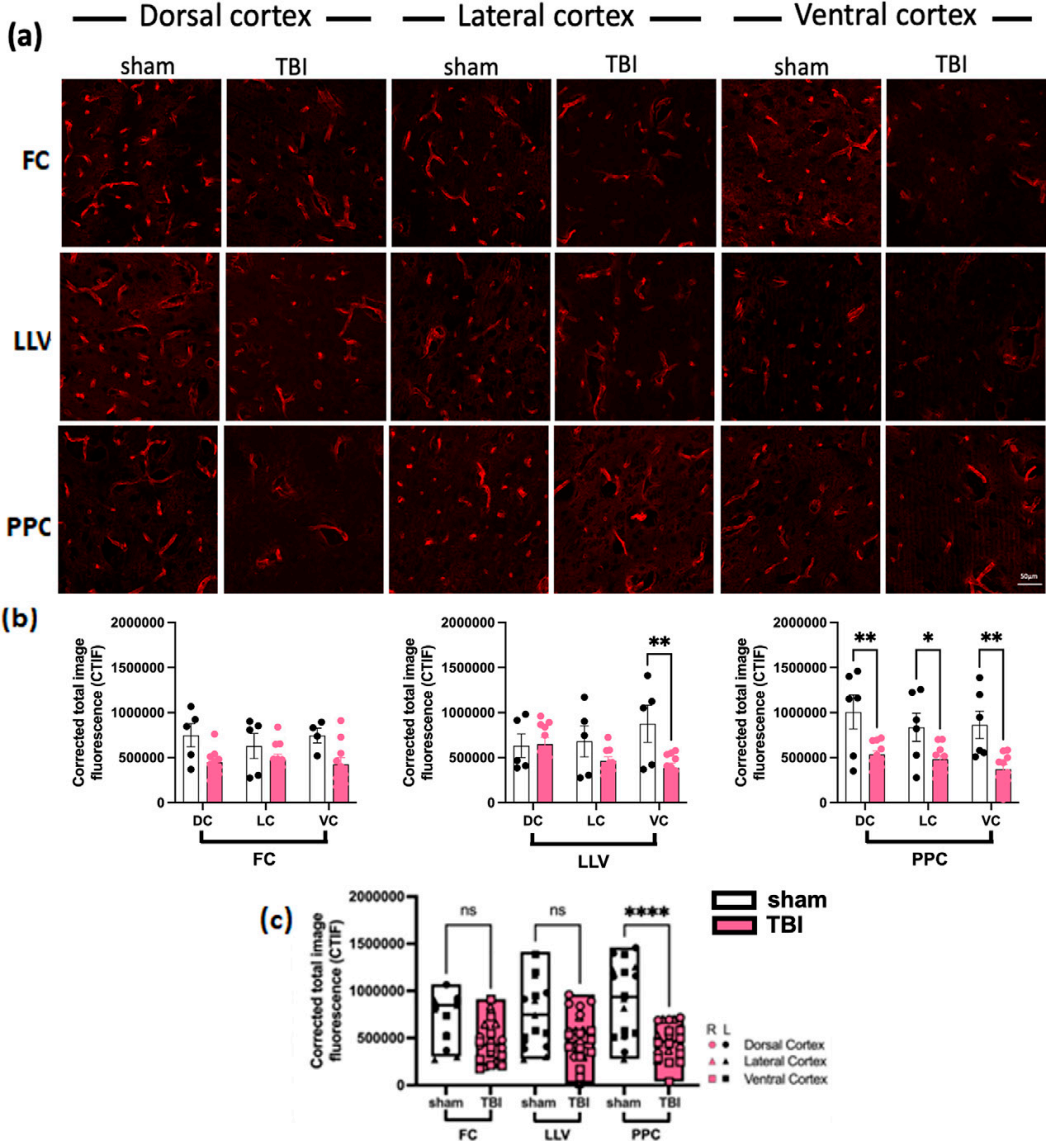
Aquaporin-4 (AQP4) is reduced after traumatic brain injury (TBI). **(a)** AQP4 staining across coronal levels corresponding to near-infrared (NIR) measurements: frontal cortex (FC, bregma +4.3 mm), left lateral ventricle (LLV, bregma +1 mm), and posterior parietal cortex (PPC, bregma −4.3 mm) for the dorsal cortex (DC), lateral cortex (LC), and ventral cortex (VC) for representative sham and TBI sections. Scale bar = 50 um. **(b)** Quantitative analysis of AQP4 staining for each coronal level in DC, LV, and VC (two-way ANOVA, Šídák’s multiple comparisons *post hoc* test, *p < 0.05, **p < 0.01). **(c)** Quantitative analysis of AQP4 staining for each coronal level for all cortical regions of interest (ROIs) (two-way ANOVA, Tukey’s multiple comparison *post hoc* test, ****p < 0.0001, **p < 0.005), white bars denote sham and pink bars denote TBI groups.

Since AQP4 channels play a role in fluid balance between the paravascular and interstitial spaces and thereby influence paravascular flow, we investigated whether there was change in AQP4 expression after TBI. We used brain sections from FC, LLV, and PPC to correspond to the coronal levels examined by NIR and quantified AQP4 expression from the DC, LC, and VC regions in each section (see Fig. 1). AQP4 expression, measured as total fluorescence (background corrected) between the left and right cortical regions were statistically the same across all brains and conditions, therefore, right and left sides were averaged, leaving three measures per brain per coronal section. While TBI animals showed trends in total AQP4 reduction compared with sham (Fig. 4a), the PPC was the only level of the brain that displayed a significant reduction across all ROIs (DC, LC, and VC) compared with sham brains; however, the VC exhibited significant difference between TBI and sham across all coronal sections (two-way ANOVA, Šídák’s multiple comparisons *post hoc* test, *p < 0.05, **p < 0.01) (Fig. 4b). When all measurements within a coronal level were combined, the PPC was the only level that demonstrated significant differences between TBI and sham conditions, with less AQP4 expression following TBI (two-way ANOVA, Tukey’s multiple comparison *post hoc* test, ****p < 0.0001, **p < 0.005) (Fig. 4c). Note that while the reduction in AQP4 expression 24 h following TBI is primarily in the posterior of the brain, the majority of CSF flow reduction as measured by NIR is anterior to AQP4 changes.

### Closed-head TBI blocks NIR dye drainage from the brain to the CLNs and leads to blood accumulation in the CLNs

**FIG. 5.**
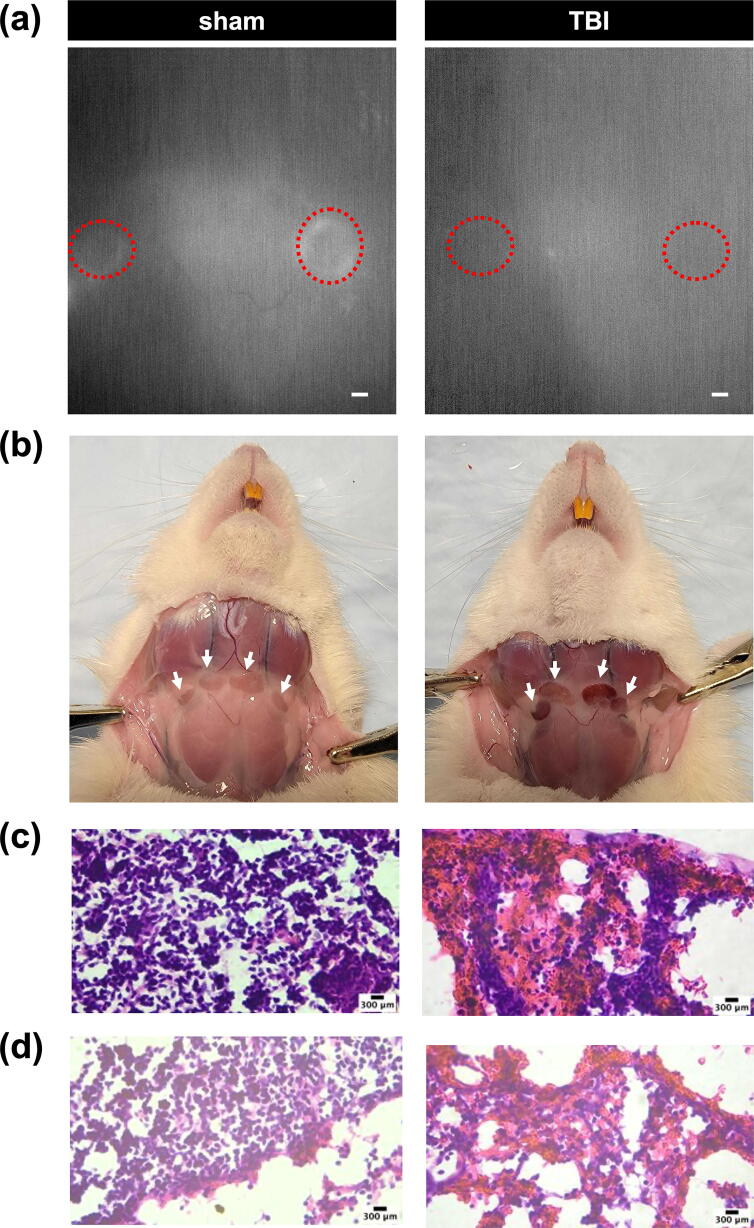
Cervical lymph nodes in traumatic brain injury (TBI) animals collect blood but not near-infrared (NIR) dye. Imaging of representative superficial cervical lymph nodes (CLNs) shows corresponding **(a)** NIR dye uptake and **(b)** blood accumulation in representative sham (left) and TBI (right) animals. The dotted red circles in **(a)** and the white arrows in **(b)** highlight the sCLNs. Scale bar = 1 mm. **(c)** Representative hematoxylin and eosin (H&E) images of dCLN in sham (left) and TBI (right) animals. **(d)** Representative H&E images of sCLN in sham (left) and TBI (right) animals. Pink denotes cytoplasm or extracellular matrix from eosin, purple denotes nuclei from hematoxylin, and red/fuchsia denotes blood (see arrows). dCLN, deep cervical lymph node; sCLN, superficial cervical lymph node.

Given the observed effect of TBI on glymph kinetics, we next determined if we could detect any changes immediately downstream of the glymphatic drainage from the brain by imaging CLNs. NIR imaging revealed the presence of NIR dye in the sCLNs in the sham animals but not the TBI animals, implying impaired function of glymphatic clearance (Fig. 5a). In addition, visual examination of the exposed sCLNs revealed blood accumulation in all of the TBI animals, but not the sham animals (Fig. 5b). After perfusion, we isolated the dCLNs and observed the same phenotype, namely, presence of blood, in all dCLN from TBI animals, but not in sham animals. Trace blood was apparent in sham and axillary LNs.

To verify the presence of blood in the CLNs, we collected sCLNs and dCLNs after sacrifice and used H&E histological staining, demonstrating presence of significant blood accumulation in the TBI LNs (Fig. 5c and d). We found that all the TBI dCLNs (Fig. 5c) and sCLNs (Fig. 5d) had blood around the edges and/or within the tissue. Rats had various numbers of sCLN and we observed a varying degree of blood in the nodes. Trace blood was also present in the sham control nodes (Fig. 5c and d) and axillary LNs (not shown). These results suggest that following TBI extravascular blood travels through the glymphatic system to the cervical lymphatics.

## Discussion

We investigated real-time intracerebral CSF kinetics in the brain 24 h after a repetitive closed-head TBI in rats and demonstrated that non-invasive *in vivo* NIR imaging is a useful tool to measure glymphatic influx and efflux dynamics. We showed spatial and temporal alterations in CSF/glymphatic perfusion throughout the brain. There was a significantly increased appearance time to the FC and APC and reduced transport time to the posterior of the brain in TBI brains compared with sham conditions. Furthermore, TBI was associated with a stark reduction in glymphatic efflux to the CLNs and blood accumulation in the CLNs.

Our results demonstrating an overall decrease of glymph flux in the brain are consistent with literature.^21,23,24^ A moderate-to-severe TBI study reported reduced uptake of fluorescently labeled ovalbumin after TBI compared with control at 1, 3, 7, and 28 days.^21^ Other pre-clinical TBI studies have demonstrated glymphatic flow impairments at 18 h post-TBI,^22^ 24 h post-TBI,^21,23^ 21 days,^45^ 28 days,^21^ and 10 weeks post-TBI.^24^ TBI biomarkers in the serum following a single closed-head impact in the mouse were significantly reduced 18 h post-TBI when the glymphatic system was disrupted,^22^ suggesting that glymphatic clearance is critical for removal of TBI-associated waste.

Glymph movement alterations following TBI may be due to fluid flow impairment axially in the paravascular spaces or altered flow between the paravascular and interstitial compartments.^46,47^ The paravascular space facing the brain parenchyma is lined by astrocytic endfeet expressing AQP4 channels, which are important for water flux across the astrocyte-paravascular space and CSF circulation.^48,49^ In the current study we provide evidence of reduced AQP4 expression in the posterior and ventral regions of the brain after TBI, which corresponds to areas with relatively higher NIR dye intensity, suggesting increased paravascular volume and accumulation of CSF (i.e., fluid stagnation).^50^ The loss of AQP4 polarization to the astrocytic endfeet is associated with disrupted glymphatic function,^49,51,52^ by reducing CSF transport into the brain parenchyma and/or ISF efflux to the paravascular spaces.^53^ Studies have shown increased paravascular volume following human mild TBI,^54,55^ supporting observations in experimental TBI. Although our current study and other colleagues have shown a decrease of AQP4 expression,^56–59^ some studies have also shown an increase of AQP4 expression after TBI.^60–63^ The difference in these findings may be due to the various injury models to induce TBI and time points after injury. Further studies are needed to elucidate the relationship between AQP4 expression and glymphatic disruption after TBI.

NIR is a non-invasive technique with a wider imaging field, reduced scattering, and enhanced skull and tissue penetration depth compared with other fluorescent imaging methods.^64–66^ PEGylated NIR dyes are ideal for assessing glymphatic flow movement, as they do not adhere to tissue or are they phagocytosed by macrophages.^18^ Several other imaging modalities have been used to visualize glymphatic flow,^67^ including two-photon microscopy,^12^ computed tomography,^68^ MRI,^24^ PET, and lower wavelength fluorescence techniques. Lower wavelength fluorescence imaging, in particular, requires surgical exposure of the area of interest due to the shallow penetration depth. MRI and PET are not widely available in the pre-clinical research setting and have significant costs associated with use. Our NIR technique is set to improve on these challenges while also offering an additional non-invasive alternative. While NIR II has been used to investigate glymphatic function following a stab wound,^69^ this is the first report to our knowledge of NIR imaging of CSF flow in experimental TBI.

We also found that NIR dye reaches the CLNs that are downstream from the brain in sham animals and that TBI caused a stark reduction in tracer efflux to the CLNs. Increased paravascular volume may contribute to reduced clearance of tracers.^70^ Although there is evidence that CSF drains into the CLNs, the mechanism of CSF entry into LNs is not fully understood.^19,71,72^ It has been shown that meningeal lymphatic drainage function is impaired by TBI and that pre-existing lymphatic disruption increases neuroinflammation and neurobehavioral outcome.^73^ It has been demonstrated that mild TBI can have a long-lasting effect on meningeal lymphatic drainage contributing to cerebral edema,^73^ which, if untreated, can damage the surrounding tissue,^74^ further underlining the importance of a normally functioning brain waste clearance system. Although there is evidence that lymphatic drainage is crucial for brain homeostasis, its role in TBI pathogenesis is still unfolding.^10,32–35^

Interestingly, we observed blood accumulation in the dCLNs and sCLNs in TBI animals, suggesting that blood from injury-induced bleeding (e.g., hematoma, hemorrhage) follows the same efflux pathway as waste and fluid described by glymphatic dynamics. Although intracranial hemorrhage has long been observed in patients,^75^ and in animals with TBI,^76,77^ observation of blood clearance through the glymphatic (i.e., extravascular) pathway, to our knowledge, has never been shown following TBI. This observation warrants further investigation and has implications for monitoring of brain bleeding and augmenting blood clearance. Not only could CLN imaging inform about brain hemorrhage but it also supports a role for the glymphatic system in clearing blood. Blood injected to the brain parenchyma to simulate hemorrhage has been observed to be cleared to the CLNs,^78^ supporting this possibility. More recently, intraventricular injection of blood has been observed to also accumulate in the CLN, the brain clearance of which is increased by photostimulation.^79^ Given the well-defined dark red color of the LNs and corroborating histological evidence, we provide these results as evidence of the glymphatic clearance of blood following closed-head TBI and point to the promise of enhanced glymphatic clearance as a therapeutic target for TBI-related intracerebral bleeding.

### Limitations

Utilizing NIR allowed us to quantify the effect of a closed-head mild TBI on glymphatic/lymphatic function, yet there are limitations to the study. Anesthesia is known to affect glymphatic function. Dexmedetomidine is known to enhance glymphatic flow by blocking norepinephrine release producing slow wave, delta oscillations.^13,80,81^ These studies were done in the dark cycle of the day (equivalent to human awake period), when glymphatic efflux is less than during the light cycle (equivalent to human sleep period) where previous reports show circadian rhythm and time of day play a role in glymph influx and clearance peaking mid-day.^82,83^ Notably, this study included only male rats, thus we are unable to infer anything about the effect of biological sex in CSF/glymph kinetics. Additional studies need to be conducted that include both male and female rats in order to investigate potential sex-based differences. Additionally, further studies are needed to expand the glymphatic flux time course due to secondary injury responses in the subacute and chronic phase of TBI pathology, which may influence fluid flux out of the brain. In addition, we used a 20 kDa PEGylated dye in this study, which may limit the interpretations of transport patterns in the brain, as solute size is known to vary intracerebral fluid dynamics.^47,84,85^

## Conclusions

Overall, the glymphatic system has been recognized as vital for CSF homeostasis, maintaining protein clearance, nutrient distribution, and normal brain signaling.^14^ The glymphatic system has been shown to be modulated by aging, genetic phenotypes, sleep–wake cycle,^80,86^ cardiac pulsations, respiration, body posture,^87^ and AQP4 expression and localization.^52^ In addition to TBI, glymphatic system dysfunction is implicated in several other neurological disorders, including but not limited to epilepsy,^88^ stroke,^89^ and Alzheimer’s disease.^90^ Despite considerable research on the pathophysiology of TBI, precision therapies remain elusive.^91,92^ Restoring lymph drainage from the brain and glymphatic function is a promising and novel therapeutic target for neuropathologies.^93–95^

## Abbreviations Used

ANOVA: Analysis of Variance
APC: Anterior Parietal Cortex
AQP4: Aquaporin-4
AU: arbitrary units
CCI: Controlled Cortical Impact
CLN: Cervical Lymph Nodes
CSF: Cerbrospinal Fluid
DAPI: 4′,6-diamidino-2-phenylindole
DC: Dorsal Cortex
dCLN: Deep Cervical Lymph Nodes
FC: Frontal Cortex
GFAP: Glial Fibrillary Acidic Protein
IS: Injection Site
ISF: Interstitial Fluid
LC: Lateral Cortex
LLV: Left Lateral Ventricle
LN: Lymph Nodes
MRI: Magnetic Resonance Imaging
NIR: Near Infrared
OCT: Optimal Cutting Temoerature
PET: positron emission tomography
PPC: Posterior Parietal Cortex
ROI: Region of Interest
ROUT: Robust Regression and OUTlier removal sCLN Superficial Cervical Lymph Node
SD: Sprague Dawley
TBI: Traumatic Brain Injury
VC: Ventral Cortex
